# Frustrated Synchronization of the Kuramoto Model on Complex Networks

**DOI:** 10.3390/e26121074

**Published:** 2024-12-09

**Authors:** Géza Ódor, Shengfeng Deng, Jeffrey Kelling

**Affiliations:** 1 Institute of Technical Physics and Materials Science, HUN-REN Centre for Energy Research, P.O. Box 49, H-1525 Budapest, Hungary; 2 School of Physics and Information Technology, Shaanxi Normal University, Xi’an 710062, China; gitsteven@gmail.com; 3 Institute for Radiation Physics, Helmholtz-Zentrum Dresden–Rossendorf, P.O. Box 510119, 01314 Dresden, Germany; j.kelling@hzdr.de; 4 Faculty of Natural Sciences, Chemnitz University of Technology, Straße der Nationen 62, 09111 Chemnitz, Germany

**Keywords:** synchronization, Kuramoto, criticality, spectral dimension

## Abstract

We present a synchronization transition study of the locally coupled Kuramoto model on extremely large graphs. We compare regular $40^{5}$ and $100^{4}$ lattice results with those of $12,000^{2}$ lattice substrates with power-law decaying long links (ll). The latter heterogeneous network exhibits $d_{s}>4$ spectral dimensions. We show strong corrections to scaling and mean-field type of criticality at d=5 , with logarithmic corrections at d=4 Euclidean dimensions. Contrarily, the ll model exhibits a non-mean-field smeared transition, with oscillating corrections at similarly high spectral dimensions. This suggests that the network heterogeneity is relevant, causing frustrated synchronization akin to Griffiths effects.

## 1. Introduction

Synchronization phenomena are abundant in nature, ranging from brain science [1] to power grids [2]. Describing them via toy models has a long history; for recent reviews, see [3,4]. One of the simplest and most well known is the one introduced by Kuramoto [5]. In its original form, it is a fully coupled system defined on a full graph. The synchronization transitions of locally coupled versions were studied on finite-dimensional lattices in [6]. In homogeneous systems, the Kuramoto model exhibits equal critical and lower critical dimensions $d_{c}=d_{l}=4$ , meaning that real phase transition can happen above $d\geq d_{l}=4$ , and is of mean-field type, meaning that it exhibits the scaling of a system defined on a fully connected graph. The entrainment transition of frequency variables can be non-mean-field-like for 2<d<4 .

On heterogeneous random graphs, the phase transition remains mean-field-like [7] according to the annealed heterogeneous mean-field approximation; however, care must be taken even in dense-network systems, particularly in the disordered phase [8]. By studying the dynamical scaling of Kuramoto on Erdos Rényi graphs, non-monotonic corrections to scaling hinder the ability to clearly see the mean-field criticality, even for very large systems [9]. Note that the disorder here was different from other `disordered’ Kuramoto models studied in the literature, where the disorder arises from independent random positive and negative couplings that add frustration to the system and can lead to glassy dynamics [10].

In lower-dimensional simplicial complex model of manifolds complexes [11] and on hierarchical modular networks, a so-called frustrated synchronization transition has been reported for spectral dimension $d_{s}<4$ [11,12]. This is similar to Griffiths phases [13] in condensed matter physics, which are caused by rare region effects and result in continuously changing dynamical scaling exponents. In [14], it was shown that the synchronized phase can only be thermodynamically stable for spectral dimensions above four, and that the phase entrainment of the oscillators can only be found for spectral dimensions greater than two. Very recently, [15] studied one-dimensional long-range random ring networks in which any two nodes on the network are connected with a probability proportional to a power-law of the distance between the nodes, and confirmed the results of [14] for the Kuramoto model.

On a large weighted human connectome network containing 804,092 nodes with topological dimension d<4 , a real synchronization phase transition is not possible in the thermodynamic limit; nonetheless, [16] was able to find a transition between partially synchronized and desynchronized states, with non-universal coupling dependent dynamical scaling. On the other hand, on the infinite-dimensional “2dll” random long link model of lateral size L=6000 Kuramoto calculations, starting from states with oscillators of fully random phases, the growth exponent of the global synchronization defined by Equation (4) at the synchronization transition point was found to be η=0.55(10) , which is away from the mean-field expectations by scaling relations η=0.75 [17]. It has been suspected that finite-size scaling and corrections hid the true mean-field behavior numerically in both [16] and [17]. Here, we provide a more extended numerical study with the aim of clarifying this issue.

## 2. Materials and Methods

The model introduced by Kuramoto [5] is one of the most studied for oscillatory systems. In addition to the original global coupled system, we can define a locally coupled version on graphs [3], in which phases $\theta_{i}(t)$ located at N nodes of a network follow the dynamical equation

(1) $${\dot{\theta }}_{i}(t)=\omega_{i}^{0}+\frac{K}{k_{i}}\sum_{j}A_{ij}\sin\left[\theta_{j}(t)-\theta_{i}(t)\right].$$

The global coupling K (divided by the node degree $k_{i}$ of the i -th node) is the control parameter, by which it is possible to tune the system between asynchronous and synchronous states. The summation is performed over the nearest neighboring nodes, with connections described by the adjacency matrix $A_{ij}$ and $\omega_{i}^{0}$ denoting the quenched self-frequency of the i -th oscillator, which is chosen from a Gaussian distribution with zero mean and unit variance. When $A_{ij}$ describes a full graph, the dynamical behavior is of mean-field type [18]. The critical dynamical behavior has been explored on various random graphs [16,17]. In regular lattices synchronization, phase transition can happen only above the lower critical dimension $d_{l}^{-}=4$ [6]. In lower dimensions, a true singular phase transition in the N→∞ limit is not possible, but partial synchronization emerges with a smooth crossover if the oscillators are strongly coupled.

To investigate the relaxation to the steady state, we measured

(2) $$z\left(t_{k}\right)=r\left(t_{k}\right)\exp\left[i\theta \left(t_{k}\right)\right]=1/N\sum_{j}\exp[i\theta_{j}\left(t_{k}\right)]\;,$$

where $0\leq r(t_{k})\leq 1$ gauges the overall coherence and $\theta (t_{k})$ is the average phase at discrete sampling times $t_{k}$ , which was chosen to follow an exponential growth $t_{k}=1+1.08^{k}$ in order to spare memory space.

The sets of Equation (1) were solved numerically for $10^{3}$ – $10^{4}$ independent initial conditions initialized by different $\omega_{i}^{0}$ , and random initial phases $\theta_{i}\left(0\right)$ were used. Then, sample averages for the phases and the frequencies give rise to the Kuramoto order parameter

(3) $$R\left(t_{k}\right)=\langle r\left(t_{k}\right)\rangle \;,$$

which is nonzero above the critical coupling strength $K>K_{c}$ , tends to zero for $K<K_{c}$ as $R\propto \sqrt{1/N}$ , or exhibits a growth at $K_{c}$ as

(4) $$R\left(t,N\right)=N^{-1/2}t^{\eta }f\left(t/N^{\tilde{z}}\right)\;$$

in case of an incoherent initial state, with the dynamical exponents $\tilde{z}$ and η . We also measured the variance of the frequencies

(5) $$\Omega \left(t\right)=\frac{1}{N}\sum_{j=1}^{N}{\left(\bar{\omega }\left(t\right)-\omega_{j}\left(t\right)\right)}^{2}\;,$$

which is expected to exhibit an asymptotic decay law $\Omega \left(t\right)\propto t^{-d/2}$ in the ordered phase by linear approximation [17], and has also been confirmed numerically in the nonlinear regime [17,19].

The Kuramoto differential equation system (1) was solved using the adaptive Bulirsch–Stoer stepper [20,21], which adjusts the step size and degree of function approximation to ensure a local absolute error and relative errors $\nu \leq 10^{-9}$ . We used the implementation in boost::odeint [22] with the VexCL backend [23,24] for its support of CUDA GPUs.

The node and edge information of sparse random graphs were stored using a memory layout optimized for efficient parallel evaluation of the Kuramoto computation of the interaction term. This implementation permitted the numerical treatment of large systems with $N=12,000^{2}$ nodes and containing up to $∼6\cdot 10^{8}$ edges while sampling thousands of realizations per graph type on a small GPU cluster.

### 2.1. The Power-Law Decaying Long Range Graph (Ll)

Graphs with variable topological and spectral dimensions can be generated algebraically by the addition of long-range connections with probability that decays with distance *l*, as

(6) $$p_{l}\propto \beta l^{-s},$$

parameterized by the exponent *s*; see for example [25]. We used this method by adding links to two-dimensional lattices with periodic boundary conditions and linear sizes L=12,000 . For s<4 , infinite graph dimensions are obtained; the probability of long-range links decreases as *s* increases, and for s>4 any long-range links are suppressed to simply provide the original underlying two-dimensional lattice. This construction provides the possibility of generating systems with dimensionality that varies from 2 to *∞* at s=4 by tuning β . For the spectral dimension, however, the situation is different, as we show in Section 3.1.

### 2.2. Spectral Analysis

The spectrum of the Laplacian matrix of a complex network encapsulates vital information about the network’s structural properties, including its connectedness and connectivity, its ability to synchronize or conduct diffusion processes, and its resilience against structural perturbations, among others [26]. In particular, it is related to the linearized Kuramoto equation, which describes a random walk. Such spectra have also been shown to encode the dimensionality information of a network [27]. Following [11,14], we adopt the normalized Laplacian *L* with elements

(7) $$L_{ij}=\delta_{ij}-A_{ij}/k_{i}$$

for unweighted networks, where $k_{i}$ denotes the degree of node *i*. The normalized Laplacian has real eigenvalues $0=\lambda_{1}\leq \lambda_{2}\leq \cdots \leq \lambda_{N}$ that form a spectrum, the density of which is characterized by the following scaling behavior [11,27]:

(8) $$\rho \left(\lambda \right)≃\lambda^{d_{s}/2-1}$$

for λ≪1 , where $d_{s}$ is the spectral dimension. The cumulative density is then provided by

(9) $$\rho_{c}\left(\lambda \right)=\int_{0}^{\lambda }d\lambda^{\prime }\rho \left(\lambda^{\prime }\right)≃\lambda^{d_{s}/2}.$$

Note that the smallest nonzero eigenvalue $\lambda_{2}$ , or Fiedler value, quantifies the connectivity of the network. In connected networks with finite spectral dimensions, the dimension $d_{s}$ is an overall measure for the local connectivity; as the system size *N* increases, the resulting larger network will also have more room for disconnection, giving rise to a smaller $\lambda_{2}$ . Therefore, $\lambda_{2}$ depends on both $d_{s}$ and *N*. By imposing $\rho_{c}\left(\lambda_{2}\right)=1/N$ (the cumulative density of this smallest nonzero eigenvalue over *N* eigenvalues), $\lambda_{2}$ then scales with the network size according to the following power law [14]:

(10) $$\lambda_{2}∼N^{-2/d_{s}}\;.$$

More generally, in order for the spectrum to display the power-law behavior in (8) and (9), each eigenvalue should follow similar leading-order scaling [28]:

(11) $$\lambda_{i}∼N^{-2/d_{s}}\;.$$

## 3. Results

### 3.1. Spectral Analysis Results

**Figure 1:**
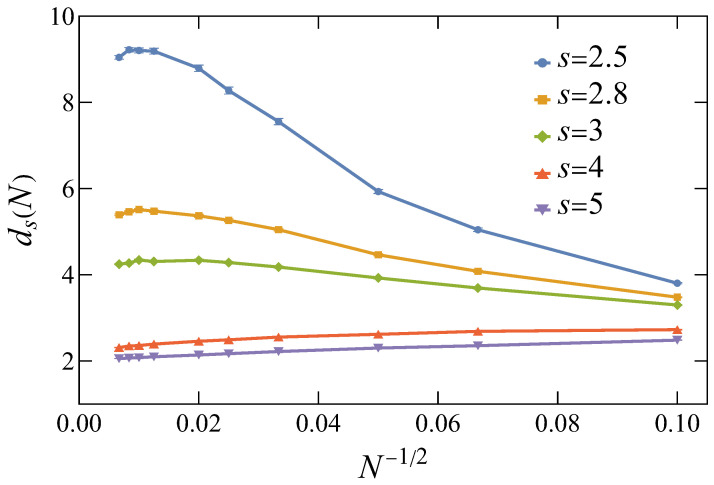
Dependence of the estimated spectral dimension $d_{s}$ on the system size *N* for s=2.5,2.8,3,4 , and 5. For each system size $N=L^{2}$ ( L=10,15,20,30,40,50,80,100,120 , and 150), Equation (10) is applied with respect to three vicinal system sizes, ${\left\lfloor L/b\right\rfloor }^{2}$ , $L^{2}$ , and ${\left\lceil bL\right\rceil }^{2}$ , to obtain $d_{s}\left(L^{2}\right)$ , where b=1.12 . All results are averaged over 4000 random realizations.

Equations (8)–(11) all suffice for estimating the spectral dimension; however, because the $d_{s}$ values obtained from Equations (8) and (9) may depend on how many low-lying eigenvalues are chosen, these two scaling relations are not very reliable. In practice, it is more appropriate to utilize the finite-size scaling relations in (10) and (11) to obtain $d_{s}$ by observing how the scaling exponents converge as N→∞ . In Figure 1, we have applied Equation (10) to estimate the dependence of $d_{s}$ on *N* for different *s* values, which shows that the scaling form in Equation (10) only provides a consistent and reliable $d_{s}$ value by extrapolating to the thermodynamic limit N→∞ . This also shows that the intended upper critical dimension $d_{sc}=4$ for our study corresponds to choosing s≈3 .

**Figure 2:**
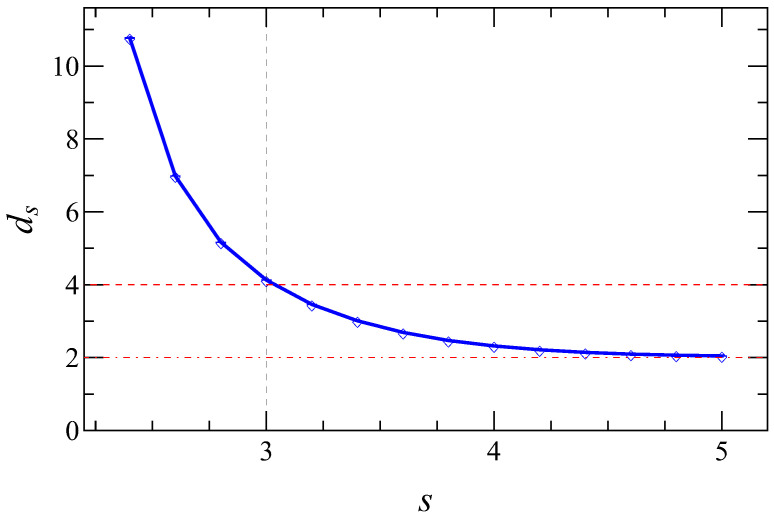
Dependence of $d_{s}$ on *s*. For each *s* value, the ten smallest non-zero eigenvalues $\lambda_{2}\cdots \lambda_{11}$ are obtained with respect to $N=140^{2},160^{2},180^{2},200^{2}$ for the large system size limit. Each eigenvalue $\lambda_{i}$ permits an estimate of $d_{s}$ according to Equation (11), averaged over 2000 realizations. The eventual estimate of $d_{s}$ for each *s* value is then obtained by averaging over these ten $d_{s}$ values. Note that the magnitudes of the error bars are hardly discernible compared to the size of the plotted points.

To more systematically determine how $d_{s}$ varies with *s* in the thermodynamic limit, we may estimate $d_{s}$ according to Equation (11) with respect to several eigenvalues for large enough system sizes. From the results shown in Figure 1, taking $N=140^{2},160^{2},180^{2}$ , and $200^{2}$ should provide reasonable estimations, while pushing the lateral size *L* to even larger values will be rather demanding in terms of computing resources for eigenvalue calculations. The resulting Figure 2 (see the caption for the calculation details) shows that $d_{s}$ decreases monotonously with respect to *s* and tends to the dimensionality of the underlying two-dimensional lattice for large *s*, as expected according to Section 2.1. This figure further justifies that $d_{s}\approx 4$ for s=3 and $d_{s}\approx 2$ for s=4 , in agreement with the relevancy of the long links. In Section 3.3, we take s≤3 , as we intend to study synchronization dynamics on networks with $d_{s}≳4$ .

### 3.2. Regular Lattice Kuramoto Order Parameter Behavior

**Figure 3:**
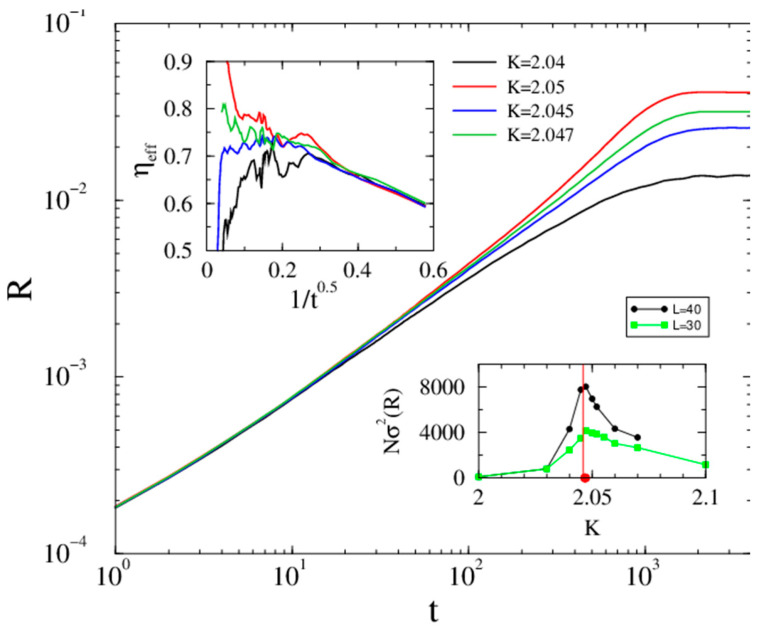
Evolution of the Kuramoto order parameter from a phase-asynchronous initial state in d=5 lattice ( $k_{i}=10$ ) of linear size L=40 near the phase transition point (coupling values ( K ) are in the legends). The top inset shows the corresponding local slopes. Bottom inset: the size-independent peaks of σ(R(K)) for L=30 and 40 mark the value of $K_{c}$ , with $K_{c}^{\prime }=K_{c}/k_{i}=0.20(1)$ as estimated in Ref. [6].

First, we solve Equation (1) in the case of five-dimensional lattices, where the mean-field type of behavior is expected. We initialized the solver by random initial phases and followed the evolution of R(t) up to $t_{max}=4000$ time steps. We performed these calculations for L=32,40 linear sizes for thousands of independent random initial conditions. Here, we show the results of L=40 in Figure 3, as for L=32 the cutoff happens at earlier times. Using a local slope analysis, defined by the logarithmic derivatives of the growth Equation (4) at the discrete time steps $t_{k}$ near the transition point

(12) $$\eta_{\mathrm{eff}}=\frac{\ln R\left(t_{k+4}\right)-\ln R\left(t_{k}\right)}{\ln\left(t_{k+4}\right)-\ln\left(t_{k}\right)}\;,$$

we estimated $K_{c}$ as well as the exponent η . After selecting a proper rescaling of the horizontal axis, a linear inflexion curve can be observed at $K=K_{c}=2.046\left(1\right)$ , separating the up- and down-veering curves corresponding to the super- and subcritical cases, respectively; also, we can extrapolate to η=0.77(2) in the t→∞ limit. The initial growth $\propto t^{1/2}$ behavior, similar to [17], leads to a strong corrections to scaling. Note that a simple power-law fit on R(t) would provide a smaller growth exponent. Our results are the first to clearly show the expected asymptotic mean-field scaling; previous analyses on smaller sizes were unable to see it due to the corrections and early time cutoff. The bottom inset of Figure 3 shows the standard deviations of the Kuramoto order parameter σ(R(K)) in the steady state, with a peak at K=2.047 , agreeing well with the $K_{c}$ , where the inflexion curve happens.

**Figure 4:**
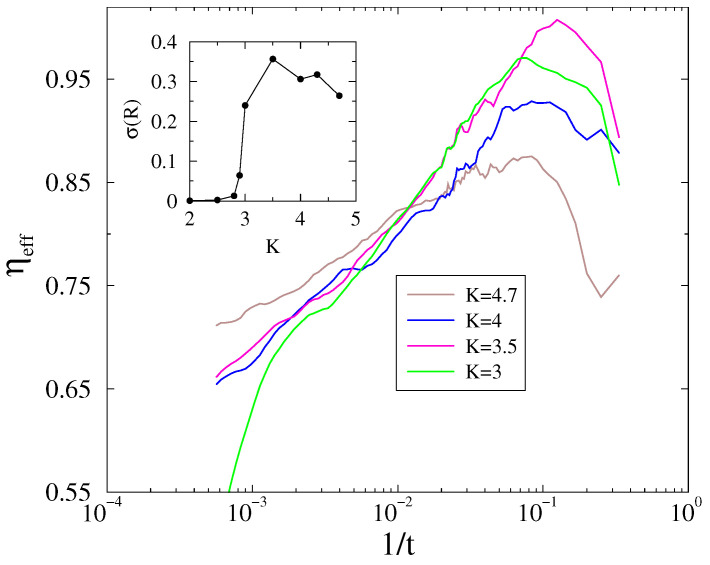
Local slopes of the evolution of the Kuramoto order parameter from a phase-asynchronous initial state in d=4 lattice of linear size L=100 near the phase transition point (coupling values (*K*) are in the legends). We plot the slopes, as logarithmic corrections to scaling are expected. Inset: σ(R(K)) peak coincides with Kc .

We repeated this analysis for d=4 , except here the expected logarithmic corrections at $d=d_{c}$ make the scaling even more complex. We show only the effective exponents for the calculations of $100^{4}$ lattices on Figure 4, again after a sample average of 1000–5000 independent initial conditions. By rescaling the horizontal axis on the logarithmic scale, an inflexion curve can be seen at $K_{c}≃3.5\left(5\right)$ , which may indicate the expected behavior at $d=d_{c}$ ; however, care must be taken due to the unknown finite-size cutoff. The inset of Figure 4 also shows the standard deviations σ(R(K)) in the steady state, with a peak at K=3.5 , agreeing well with a $K_{c}$ , where the growth inflexion curve happens. Thus, we only claim that our numerical results can confirm the mean-field behavior, with logarithmic corrections.

### 3.3. The Ll Graph Order Parameter Results

**Figure 5:**
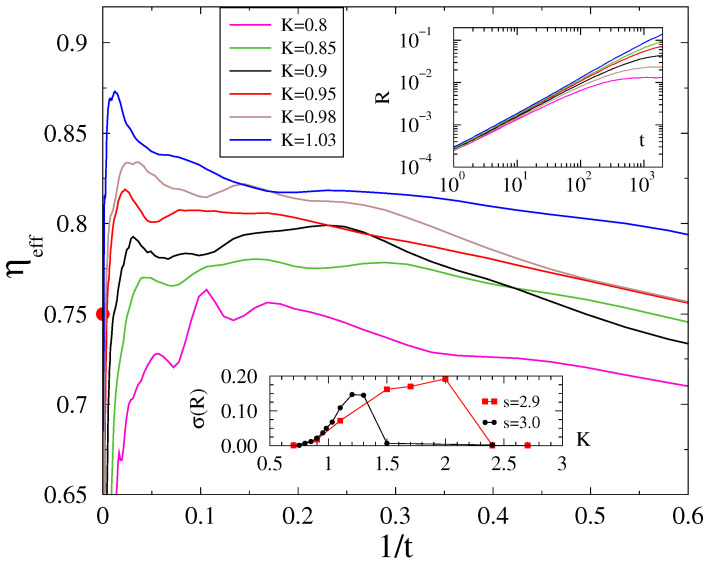
Local slopes of the evolution of the Kuramoto order parameter from a phase-asynchronous initial state on the ll graph with s=3 and linear size L=12,000 near the phase transition point (coupling values (*K*) are in the legends). Top inset: R(t) of the same data. Bottom inset: σ(R(K)) for s=2.9,3.0 with fluctuation peaks away from $K_{c}$ .

Now we show our results for the ll graph, where we used large two-dimensional base lattices with maximum linear sizes L=12,000 and added power-law (PL) decaying random long links with exponent s=3 , corresponding to $d_{s}\geq 4$ , according to the spectral analysis shown in Section 3.1. Thus, we would expect to see a simple mean-field behavior. Instead, as Figure 5 shows, we find a smeared transition, without any clear separatrix extrapolating to the mean-field value η=0.75 in the infinite time limit. Unlike in the regular lattice cases, the local slope curves seem to saturate in the region 0.85<K<0.98 and 0.3<1/t<0.03 before the finite-time cutoff. However, the curves also exhibit wavy modulations which persist even after averaging over 2000 independent samples, which prohibits extending the calculations any further.

Such wavy modulations and non-monotonic corrections in the local exponents were also present in the Kuramoto case on random Erdos–Rényi graphs [9], where the authors could not clearly justify relevant deviations from the mean-field behavior. We also calculated the standard deviations of the Kuramoto order parameter in the steady state, which takes the maximal value in case of regular lattices at $K_{c}$ . As the bottom inset of Figure 5 shows, σ(R(K)) exhibits a fluctuating peak at K≃1.25 , well above K=0.8 , where mean-field dynamical scaling might happen. This is very similar to Griffiths phases, where one finds extended fluctuation regions away from the critical point. Solving Kuramoto on ll graphs with smaller *s* values runs into rapidly increasing computational difficulties, as it requires larger memory and the communication via long links becomes slow.

**Figure 6:**
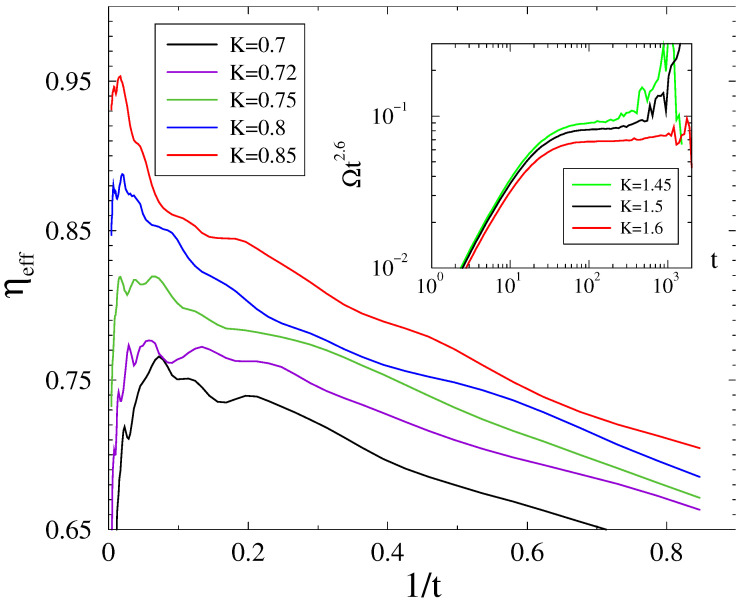
Local slopes of the evolution of the Kuramoto order parameter from a phase-asynchronous initial state on the ll graph with s=2.9 and linear size L=12,000 near the phase transition point (coupling values (*K*) are in the legends). The inset shows the evolution of the frequency spread $\Omega \left(t\right)t^{2.6}$ for s=2.8 and L=6000 .

We also obtained results for s=2.9 , resulting in a higher spectral dimension and where the deviations from the mean-field are even more obvious, as shown on Figure 6.

Although we have obtained results for a limited number of couplings and the corrections are wavy, our results suggest an asymptotical dynamical critical behavior for $0.7<K_{c}<0.8$ , with a *K*-dependent exponent η>0.75 , larger than the mean-field value. Again, the steady state fluctuations σ(R(K)) exhibit extended smeared behavior above the $K_{c}$ region with a peak at K≃2 , as can be seen in the bottom inset of Figure 5.

For s=2.8 , the scaling region of R(t) is even more narrow; thus, the inset of Figure 6 instead shows the evolution of the frequency spread multiplied by $t^{2.6}$ in a system with with L=6000 , for which the PL decay can be found in case of K=1.6 . The linear approximation predicts $\Omega \left(t\right)\propto t^{-d/2}$ for Euclidean lattices below $d_{c}$ [17]. The observed behavior agrees well with the spectral dimension $d_{s}\approx 5$ in case of s=2.8 , and again contradicts with the mean-field behavior $\Omega \left(t\right)\propto t^{-2}$ .

## 4. Discussion

In conclusion, we have compared the synchronization transition of the Kuramoto model at and above the upper critical dimension, finding numerical evidence that the quenched topological disorder is relevant and that a smeared frustrated phase transition seems to emerge instead of the mean-field behavior. This appears even more clearly on lattices of the $d_{s}\geq 4$ spectral dimensions. However, we note that an oscillating correction to scaling is also present in the effective exponents, which makes it more difficult to analyze data even when considering very large sizes and averages over thousands of independent initial conditions. Previously, such oscillations have been shown in earlier numerical results of the dynamical Kuramoto model [17] on both lattices and random graphs [9]. To show the dimensional equivalence, we determined the spectral dimension on ll graphs, although we note that the topological and graph dimensions are expected to be equal on regular graphs. The graph dimension of the s≤3 ll graphs considered here is infinite. The existence of a frustrated phase transition at high dimensions is crucial for understanding models of the brain, and warrants further investigations in this direction.

## Data Availability

Raw data are available from the authors on request.
