# Correction: Ódor et al. Frustrated Synchronization of the Kuramoto Model on Complex Networks. *Entropy* 2024, *26*, 1074

**DOI:** 10.3390/e28020203

**Published:** 2026-02-11

**Authors:** Géza Ódor, Shengfeng Deng, Jeffrey Kelling

**Affiliations:** 1 Institute of Technical Physics and Materials Science, HUN-REN Centre for Energy Research, P.O. Box 49, H-1525 Budapest, Hungary; 2 School of Physics and Information Technology, Shaanxi Normal University, Xi’an 710062, China; gitsteven@gmail.com; 3 Institute for Radiation Physics, Helmholtz-Zentrum Dresden–Rossendorf, P.O. Box 510119, 01314 Dresden, Germany; j.kelling@hzdr.de; 4 Faculty of Natural Sciences, Chemnitz University of Technology, Straße der Nationen 62, 09111 Chemnitz, Germany

## Text Correction

There was an error in the original publication [1]. In Equation (1), the global control parameter K should be divided by the node degree k in all calculations, which provides an overall shift of the synchronization point $K_{c}$ by a factor of 10 in case of d=5 lattices. To compensate for it, we added a division factor $k_{i}$ to Equation (1), which makes the results in d=5 comparable with former numerical findings.

A correction has been made to Materials and Methods, paragraph 1:

“The model introduced by Kuramoto [5] is one of the most studied for oscillatory systems. In addition to the original global coupled system, we can define a locally coupled version on graphs [3], in which phases $\theta_{i}(t)$ located at N nodes of a network follow the dynamical equation

(1) $${\dot{\theta }}_{i}(t)=\omega_{i}^{0}+\frac{K}{k_{i}}\sum_{j}A_{ij}\sin\left[\theta_{j}(t)-\theta_{i}(t)\right].$$

The global coupling K (divided by the node degree $k_{i}$ of the i-th node) is the control parameter, by which it is possible to tune the system between asynchronous and synchronous states. The summation is performed over the nearest neighboring nodes, with connections described by the adjacency matrix $A_{ij}$ and $\omega_{i}^{0}$ denoting the quenched self-frequency of the i-th oscillator, which is chosen from a Gaussian distribution with zero mean and unit variance. When $A_{ij}$ describes a full graph, the dynamical behavior is of mean-field type [18]. The critical dynamical behavior has been explored on various random graphs [16,17]. In regular lattices synchronization, phase transition can happen only above the lower critical dimension $d_{l}^{-}=4$ [6]. In lower dimensions, a true singular phase transition in the N→∞ limit is not possible, but partial synchronization emerges with a smooth crossover if the oscillators are strongly coupled.”

## Upgraded Figure

There is an update to Figure 3 in the original publication as published. “To check if there is a real phase transition with a critical point $K_{c}$ independent of the system size, the vertical axis for the inset of the fluctuation σ(R) is rescaled with respected to the system size N, which shows the expected FSS in 5d.” The corrected Figure 3 appears below.

The authors state that the scientific conclusions are unaffected. This correction was approved by the Academic Editor. The original publication has also been updated.

## Figures and Tables

**Figure 3:**
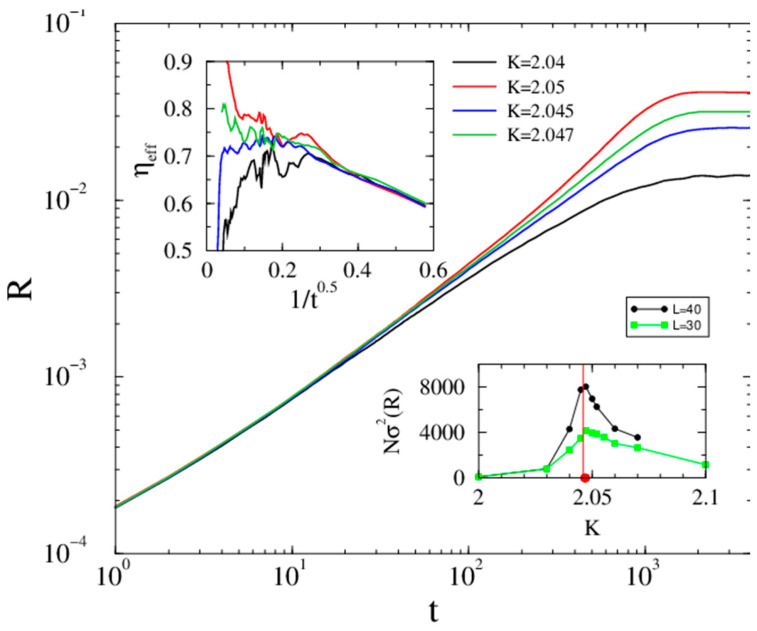
Evolution of the Kuramoto order parameter from a phase-asynchronous initial state in d=5 lattice ($k_{i}=10$) of linear size L=40 near the phase transition point (coupling values (K) are in the legends). The top inset shows the corresponding local slopes. Bottom inset: the size-independent peaks of σ(R(K)) for L=30 and 40 mark the value of $K_{c}$, with $K_{c}^{\prime }=K_{c}/k_{i}=0.20(1)$ as estimated in Ref. [6].
